# Supplementary material for: Cardio-Respiratory Events and Food Autonomy Responses to Early Uni-Modal Orofacial Stimulation in Very Premature Babies: A Randomized, Controlled Study
Source: Children (Basel). 2021 Dec 16;8(12):1188. doi: 10.3390/children8121188 (PMC8700206; doi:10.3390/children8121188)
Supplement: Supplementary file 1 [file children-08-01188-s001.zip › children-1456643-supplementary.pdf]

Supplemental Table S1. Nutritional follow-up at 6 months, corrected age, and children's QUALIN quality of life score at 6 and 12 months of age, as assessed by parents (experimental vs. control group).

**Follow-up**	**With OFS (*n* = 15)**	**Without OFS (*n* = 14)**	**P***
Number of breast feedings at 6 months (artificial/maternal)	15 (100)/ 0	14 (100)/ 0	/
Diversification at 6 months	15 (100)	14 (100)	/
Normal gag reflex at 6 months	15 (100)	14 (100)	/
Infant's quality of life as assessed by parents			
6 months QUALIN Score	0.95 ± 0.26	0.90 ± 0.18	0.70
12 months QUALIN Score	1.06 ± 0.24	0.96 ± 0.27	0.36

Data are presented as n (%) unless stated differently; OFS: oro-facial stimulation; QUALIN Questionnaire: quality of life score for infants and very young children, comprising 34 items. Twelve-month follow-up: experimental group (n=13), control group (n=14). p: value of the difference between the groups with available data, obtained by $\chi 2$ test. *Value of p in univariate analysis. $p < 0.05$: statistically significant difference.

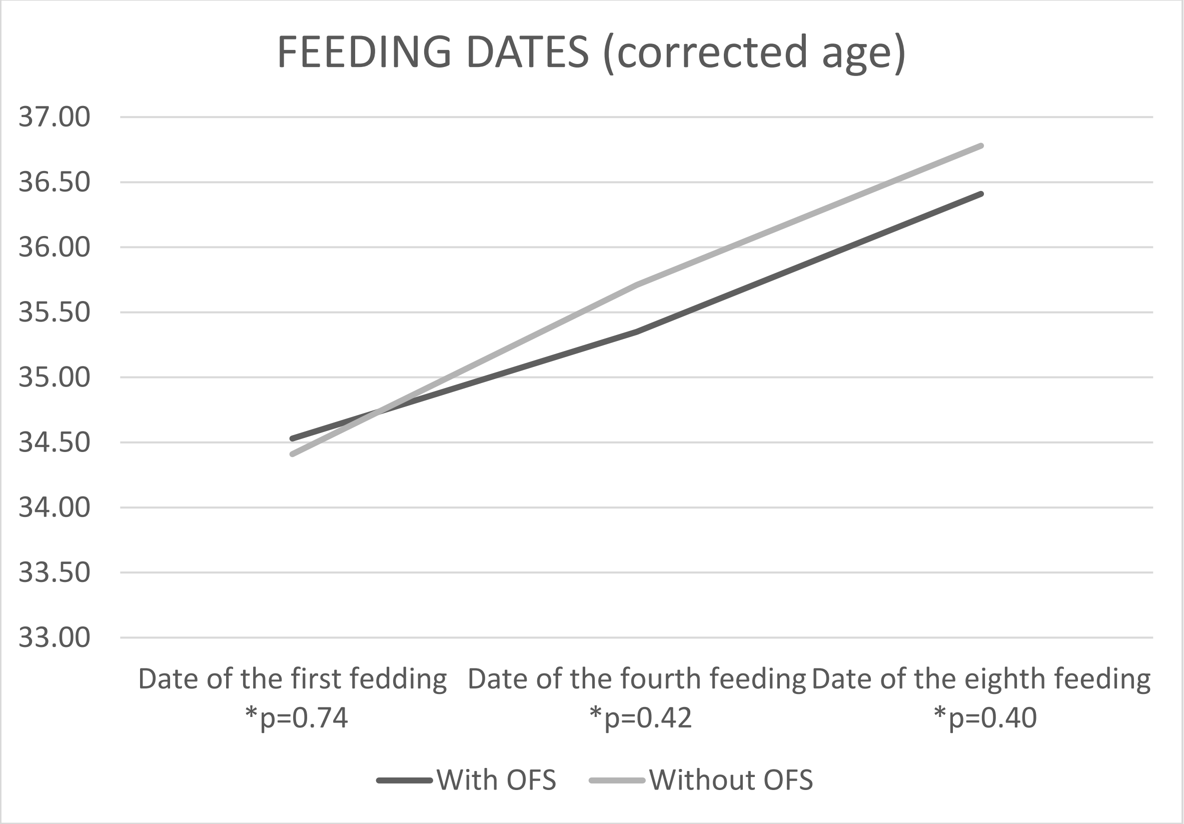

Figure S1. Experimental and control group dates of first, 4TH and 8TH independent feeds, Week GA-adjusted. Abbreviations: OFS: oro-facial stimulation. p value of the difference between the groups with available data, $p < 0.05$: statistically significant difference. * Value of p in univariate analysis.
